# Pattern of Recurrence in 428 Patients With Thoracic Esophageal Squamous Cell Carcinoma After Radical Surgery and Its Implication in Postoperative Radiotherapeutic Clinical Target Volume

**DOI:** 10.3389/fonc.2021.652365

**Published:** 2021-04-15

**Authors:** Tiantian Cui, Hongjiao Zhang, Tao Yu, Yiru Chen, Chengxin Liu, Qian Zhao, Jian Zhu, Baosheng Li, Wei Huang

**Affiliations:** ^1^ Shandong Cancer Hospital and Institute, Shandong First Medical University and Shandong Academy of Medical Sciences, Jinan, China; ^2^ Department Seven of General Surgery, The Second Affiliated Hospital of Harbin Medical University, Harbin, China; ^3^ Department VI of Radiation Oncology, Shandong Cancer Hospital and Institute, Shandong First Medical University and Shandong Academy of Medical Sciences, Jinan, China; ^4^ Department of Radiation Oncology, Shandong Cancer Hospital and Institute, Shandong First Medical University and Shandong Academy of Medical Sciences, Jinan, China

**Keywords:** esophageal cancer, lymph nodes, postoperative radiotherapy, recurrence and metastasis, clinical target volume

## Abstract

**Purpose:**

This study aimed to analyze the recurrence patterns of thoracic esophageal squamous cell carcinoma (ESCC) after radical surgery, and to understand its implication in the clinical target volume (CTV) design of postoperative radiotherapy (PORT) in patients with ESCC.

**Methods and materials:**

A total of 428 recurrent ESCC patients after radical surgery between 2014 and 2018 were included in this study. Recurrence patterns, especially anastomotic and regional lymph node recurrence (LNR), were analyzed. A T-shaped CTV were proposed for PORT and were evaluated whether it could cover most of regional LNR.

**Results:**

These patients all experienced anastomotic and/or regional LNR. Among the 428 patients, 27 cases (6.3%) had anastomotic recurrence only, and184 cases (43.0%) had LNR only. Those sites with an LNR rate higher than 15% in upper thoracic ESCC were as follows: No.101, No.104R, No.104L, No.106recR, No.106recL, No.106pre, No.106tb, No.107, and No. 109. Those with middle thoracic ESCC were as follows: No.104R, No.104L, 106recR, No.106recL, No.106pre, No.106tb, and No.107. Lastly, individuals with lower thoracic ESCC were as follows: No.104L, 106recR, No.106recL, No. 106pre, No. 106tb, No.107, and abdominal No. 3. The proportion of LNR not included in the proposed T-shaped CTV was 12.5% (1/8), 4.7% (6/128), and 10.4% (5/48) in the upper, middle, and lower thoracic segments, respectively.

**Conclusions:**

LNR was the most common type of local-regional recurrence in patients after radical surgery. Supraclavicular, superior and middle mediastinal lymph nodes had the highest recurrence rate, the rate of LNR which was outside T-shaped PORT CTV we proposed was less than 15%.

## Introduction

Esophageal cancer, especially esophageal squamous cell carcinoma (ESCC), has a high incidence rate in China and East Asia. According to a previous study, 90% of esophageal cancer patients are diagnosed with ESCC in China (1). For patients with thoracic ESCC, esophagostomy is a highly effective treatment. Nevertheless, the recurrence rate of ESCC after radical surgery ranges as high as 40–50% (2), with locoregional recurrence being the major cause of treatment failure (3, 4). The main site of recurrence for thoracic ESCC is in the middle and lower thoracic areas. Previous studies have shown that postoperative radiotherapy (PORT) after performing a radical resection of esophageal cancer can reduce the local recurrence rate (5). However, the clinical target volume (CTV) for PORT on thoracic ESCC patients is still controversial.

Radiation oncologists have proposed that the PORT CTV includes the bilateral supraclavicular area, mediastinum, and left gastric lymph nodes, which covers most locoregional recurrence sites. However, to contour PORT CTV accurately, we must understand the pattern of postoperative recurrence in ESCC, especially the patterns of lymph node recurrence (LNR). However, a detailed study of the recurrence patterns on specific lymph node stations is lacking. Therefore, this study analyzed the recurrence patterns of ESCC patients after radical surgery and evaluated the recurrence of thoracic ESCC according to division of upper, middle and lower thoracic segments. From this, we propose a more reliable PORT CTV.

## Methods and Materials

### Inclusion and Exclusion Criteria

The 428 patients who experienced anastomotic and/or regional LNR after radical surgery at Shandong Cancer Hospital and Institute were received and retrospectively analyzed from July 2014 to December 2018. To be eligible for this study, patients needed to meet the following criteria: (1) radical esophagostomy applied to completely remove the whole tumor; (2) ESCC as confirmed by pathology; (3) radiotherapy or chemotherapy performed after surgery; (4) use of routine computed tomography (CT) scanning of the chest and abdomen to determine the specific location of the LNR; and (5) pathological stage T1-4bN0-4M0-1a. Meanwhile, the exclusion criteria for patients were the following: (1) histological diagnosis of adenocarcinoma or other types; and (2) complication with other malignant tumors. The gender, age, tumor location, pathological differentiation and recurrent lymph node stations (No. 101-114) were recorded.

### Definition Principles of Lymph Nodes

The LNR of ESCC was based on the guidelines set by the Japan Esophageal Society (JES) (6, 7). The lymph node station was defined as number 101–114 following the JES guidelines. The LNS delineation reported by Huang et al. (8) was used for reference in this study. Meanwhile, the lymph nodes were classified into five categories: cervical (No.101–104), thoracic upper mediastinum (No.105–106), thoracic middle mediastinum (No.107–109), thoracic lower mediastinum (No.110–114), and abdominal lymph nodes. Next, the sites where the recurrence rate of lymph nodes was more than 15% were counted. According to the previous study, any sites with a rate higher than 15% were considered high-risk areas and considered for inclusion in the PORT CTV for patients with locally advanced disease (9). The diagnostic methods of the LNR included examinations, B-mode ultrasound, computed tomography (CT), positron emission tomography–computed tomography (PET/CT), or histological confirmation with biopsy results, if possible. Recurrence in anastomoses was confirmed using thoracic CT, PET/CT, and esophagoscopy.

### Locoregional Recurrence and Distant Metastasis

All 428 patients with ESCC experienced anastomotic recurrence or locoregional LNR after radical surgery. Following a previous study (10), LNR was diagnosed according to the following conditions: (1) nodes reappearing after complete disappearance and (2) new nodes appearing in the regions where enlarged nodes had not existed before. Distant metastasis was defined as a tumor metastasis occurring in other organs, such as the liver, lungs, or non-regional lymph nodes.

### Statistical Analysis

Clinical data were reported as a percentage, and the locoregional recurrence rate was calculated from the surgery to the time of the first recurrence.

## Results

### Patterns of Recurrence and PORT

In the 428 recurrent patients with ESCC after radical surgery, 27 patients had anastomotic recurrence (6.3%), 184 patients had LNR (43.0%), 56 patients had anastomosis with LNR (13.1%), 10 patients had distant metastasis with anastomotic recurrence (2.3%), 112 cases had distant metastasis with LNR (26.2%), and 39 cases had distant metastasis with anastomosis and LNR (9.1%) (**Table 1**). It is also worth mentioning that, of the 428 patients in this study, 289 received PORT and 135 received no PORT. However, of the 289 patients who received PORT, 97 had distant metastasis (33.6%), and 36 had an anastomosis with LNR (12.5%). Meanwhile, among the 135 patients who did not receive PORT, there were 62 cases of distant metastasis (45.9%) and 19 cases of anastomosis with LNR (14.1%) (**Table 1**).

**Table 1:** Recurrence patterns and adjuvant PORT of the 428 patients with ESCC after radical surgery.

	Characters	Number of cases (%)
	AR only	27 (6.3)
	LNR only	184 (43.0)
**Recurrence patterns**	AR + LNR	56 (13.1)
	DM + AR	10 (2.3)
	DM + LNR	112 (26.2)
	DM + AR+ LNR	39 (9.1)
	**Total**	**428 (100)**
	With PORT	289 (67.5)
	DM	97 (33.6)
	AR + LNR	36 (12.5)
**Adjuvant PORT**	Without PORT	135 (31.5)
	DM	62 (45.9)
	AR + LNR	19 (14.1)

AR, anastomotic recurrence; LNR, lymph node recurrence; DM, distant metastasis.

### LNR

**Figure 1:**
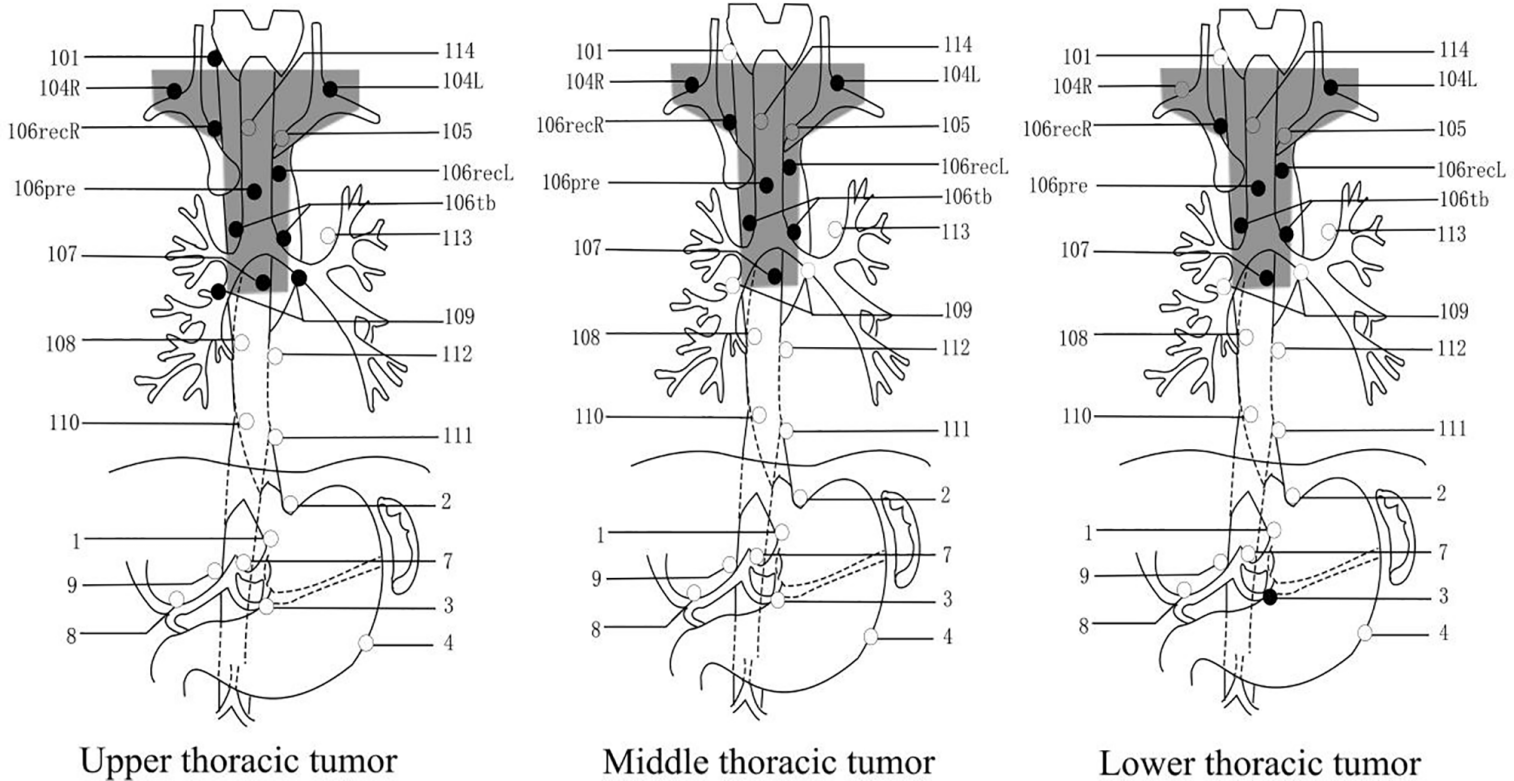
The lymph nodes in different locations with thoracic ESCC. Caption: In the above three pictures, the black dots are the location of the LNR probability of more than 15%. The shaded parts of the box are the proposed T-shaped CTV areas.

The most common pattern of recurrence was LNR, which was found in 184 patients (43.0%). A total of 184 cases had ESCC. The LNR was located in the upper thoracic esophagus (Ut) in 8 patients (4.3%), in the middle thoracic esophagus (Mt) in 128 patients (69.6%), and in the lower thoracic esophagus (Lt) in 48 patients (26.1%). The lymph node stations with a recurrence rate of the more than 15% in patients with upper thoracic tumors were No. 101, No. 104R, No. 104L, No. 106recR, No. 106recL, No. 106pre, No. 106tb, No. 107, and No. 109. The lymph node stations with a recurrence rate of the more than 15% in patients with middle thoracic tumors were No. 104R, No. 104L, No. 106recR, No. 106recL, No. 106pre, No. 106tb, and No. 107. Lastly, the lymph node stations with a recurrence rate of the more than 15% in patients with lower thoracic tumors were No. 104L, No. 106recR, No. 106recL, No. 106pre, No. 106tb, No. 107, and abdominal No. 3. **Table 2** and **Figure 1**. show the recurrence rate of lymph nodes in different locations. In summary the No.104, No.106, and No.107 lymph node stations had recurrence rates higher than 15%.

**Table 2:** The LNR rate in different locations of thoracic ESCC.

No	Ut		Mt		Lt	
	Location	Cases (%)	Location	Cases (%)	Location	Cases (%)
1	101	2/8 (25.0)	101	9/128 (7.0)	101	3/48 (6.3)
2	104R	2/8 (25.0)	104R	24/128 (18.8)	104R	6/48 (12.5)
3	104L	2/8 (25.0)	104L	25/128 (19.5)	104L	13/48 (27.1)
4	105	1/8 (12.5)	105	7/128 (5.5)	105	3/48 (6.3)
5	106recR	4/8 (50.0)	106recR	60/128 (46.9)	106recR	19/48 (39.6)
6	106recL	3/8 (37.5)	106recL	60/128 (46.9)	106recL	19/48(39.6)
7	106pre	3/8 (37.5)	106pre	61/128 (47.7)	106pre	19/48 (39.6)
8	106tb	3/8 (37.5)	106tb	60/128 (46.9)	106tb	24/48 (50.0)
9	107	2/8 (25.0)	107	23/128 (18.0)	107	11/48 (22.9)
10	108	1/8 (12.5)	108	9/128 (7.0)	108	2/48 (4.2)
11	109	2/8 (25.0)	109	14/128 (10.9)	109	6/48 (12.5)
12	110	0	110	10/128 (7.8)	110	2/48 (4.2)
13	111	0	111	1/128 (0.8)	111	0
14	112	1/8 (12.5)	112	13/128 (10.2)	112	2/48 (4.2)
15	113	1/8 (12.5)	113	17/128 (13.3)	113	6/48 (12.5)
16	114	0	114	8/128 (6.3)	114	1/48 (2.1)
17	1	0	1	6/128 (4.7)	1	5/48 (10.4)
18	2	0	2	10/128 (7.8)	2	7/48 (14.6)
19	3	0	3	12/128 (9.4)	3	10/48 (20.8)
20	4	0	4	0	4	2/48 (4.2)
21	7	0	7	8/128 (6.3)	7	7/48 (14.6)
22	8	0	8	3/128 (2.3)	8	1/48 (2.1)
23	9	0	9	0	9	2/48 (4.2)

Ut, upper thoracic; Mt, middle thoracic; Lt, lower thoracic.

### Analysis of T-Shaped PORT CTV

We proposed a T-shaped CTV for PORT in thoracic ESCC according to the rates of LNR higher than 15% which occurred in the following stations: No.104, No.106, and No.107 (**Figure 1**). The rates and sites of LNR not included in the proposed T-shaped CTV were a 12.5% (1/8) rate found in the upper thoracic segment, a 4.7% (6/128) rate found in the middle thoracic segment, and a 10.4% (5/48) rate found in the lower thoracic segment. The proposed T-shaped PORT CTV could mostly cover the LNR sites.

## Discussion

Most patients with ESCC experience recurrence after radical resection of esophageal cancer. We retrospectively examined 428 postoperative patients in this study with ESCC to make an assay of the patterns of recurrence. Furthermore, we explored the recurrence rates and the regularity of lymph nodes in different locations of thoracic ESCC, according to the JES. We can confirm that patterns of recurrence in ESCC were attributed to the local recurrence. Additionally, a retrospective study showed that most patterns of recurrence in ESCC were local-regional in nature, and our findings are consistent with these results. For the recurrence of lymph node, we can conclude from the above results (**Table 2** and **Figure 1**) that the No.104, No.106, and No.107 lymph node stations had a recurrence rate of higher than 15%. However, there is a high incidence of recurrence in the No.109 station of the upper thoracic segment. We speculate that the reason for this is that the number of cases (8) collected in the upper part of the chest was too small. The corresponding regions of the lymph nodes in these three stations were the supraclavicular and upper-middle mediastinum. Previous research data have shown that ESCC recurrence predominantly occurs at the supraclavicular and upper-middle mediastinum regions, accounting for 80–90% of the total recurrences (10, 11); our results approximated those numbers of the previously published studies.

In addition, the number of lymph nodes dissected is also an essential factor affecting postoperative recurrence. Unfortunately, this study does not elaborate on this systematically, but previous studies have reflected on the importance of lymph node dissection. The rate of the total lymph node metastasis was as high as 57.8%, and the rate of the cervical LNR was found to be 41.6% in three-field lymphadenectomy (12). Besides this, it has been reported that in two-field lymph node dissections, 52 of the 96 patients relapsed after esophagectomy. The relapses were primarily mediastinal in 41 cases and cervical/supraclavicular in 8 cases (3). It is reported that the locoregional recurrence rate was 30% for radical resection and 60% for R1 or R2 resections. However, Katayama et al. (13) clearly illustrated that all kinds of forms of recurrence and metastasis are still very high even after a sweeping of the lymph nodes. However, it is challenging to dissect lymph nodes completely. Therefore, we should consider some effective treatment combinations in the future.

Although there is no consensus on the use of adjuvant radiotherapy in prospective studies (14), in this study, we examined the effect of PORT on recurrence. Of the 428 patients, 289 received PORT, and 135 received no adjuvant radiotherapy. The above results show that PORT can reduce the rates of distant metastasis and anastomosis with LNR, which is consistent with the results of a previous study (5). It was reported that PORT with 50–60 Gy significantly reduced the recurrence rate of 549 high-risk ESCC patients with positive lymph nodes (15). While it is clear that PORT for esophageal cancer improves the outcome in some patients, the present study does not go into great detail concerning this argument, and this specific content will be examined in the later research. However, some statistics have shown that adjuvant radiotherapy is not always beneficial (16). For PORT, we should take into consideration the importance of choosing the right procedure for each patient.

Currently, the scope of the PORT CTV of thoracic ESCC is still controversial. Nevertheless, the proposed target volumes in earlier studies have generally fallen into five main categories: (1) the bilateral supraclavicular areas and the whole mediastinum (17); (2) the bilateral supraclavicular areas, the entire mediastinum and the left gastric lymph nodes (18); (3) the tumor bed alone (19); and (4) a T-shaped field including the bilateral lower cervical, supraclavicular areas, and the upper portion of the mediastinum (15); (5) 5–8 cm outside the tumor bed vertically and 2 cm horizontally without prophylactic irradiation of bilateral supraclavicular areas (20).

In this study, we also put forward our own views on the PORT CTV of ESCC. There are abundant lymph nodes in the submucous membrane of the esophagus, which makes it more difficult for us to define the CTV. For thoracic ESCC, the lymphatic vessel drainage could be set into any of the three-fields; however, there is usually one predominant area of drainage, which depends on the tumor’s location (21). We suggest adopting a larger region to cover the area of high-risk lymph nodes. From **Table 2**, we can see the recurrence rate of lymph nodes located in different sites of thoracic ESCC. For upper thoracic ESCC, we suggest that No.104, No.106 (especially 106 recR), and No.107 be included in the CTV. Meanwhile, the recurrence pattern of middle thoracic ESCC is bidirectional: it can relapse to the upper mediastinum or to the middle and lower mediastinum and abdominal cavity. According to the above results, there was a higher recurrence rate in No. 113 and abdominal 2 and 3, but it was lower than 15%. Therefore, we suggest that middle thoracic CTV still comprises No. 104, 106, and 107, and that the packaging of other lymph nodes be set according to the specific clinical conditions. Concerning lower thoracic ESCC, one of the issues we must pay attention to is lower thoracic esophageal cancer being more likely to have an abdominal recurrence than tumors in other locations, especially abdominal 1, 2, 3, and 7. Although, our proposed CTV in this study does not include abdominal lymph nodes, it can comprehensively cover more than 85% of LNR. Our advice for the CTV in lower thoracic ESCC is that No. 104, 106, and 107 should be mainly covered. Of course, adding abdominal lymph nodes may provide better therapeutic effect for some subgroups of patients, but this still needs to be further determined.

In addition to the items mentioned above, T stage, the length of the tumor, and histologic differentiation are the main factors of lymph node recurrence (21). The above factors should be taken into account in the comprehensive formulation of CTV. We believe that CTV should be customized to each patient by experienced oncologists according to the condition of the patient’s tumor. However, if the CTV range is too small, it will miss the tumor cells; furthermore, if the radiation range is too large, it will cause unnecessary damage to the patient’s body. Given this, oncologists must balance the advantages and disadvantages, and it is essential to choose the appropriate PORT CTV.

This study has both advantages and disadvantages. The merits of this study fall into two main categories. First, all 428 patients experienced postoperative recurrence of esophageal cancer, primarily lymph node recurrence. These patients belong to a large sample of data, which can explain some problems to a certain extent. Second, in order to explore the regularity of LNR, we defined lymph node stations No. 101–114 according to the JES guidelines and delineated upper thoracic, middle thoracic, and lower thoracic segments. This way, the pattern of recurrence could be batter grasped. Meanwhile, the limitations of this study were that first, there was no control group in this study. It was therefore impossible to carry out multifactor analysis, and we could not conclusively determine the factors affecting the postoperative recurrence of ESCC. Second, an excessive concentration of data in a certain period may lead to slight deviation. Ultimately, the regularity of postoperative recurrence of esophageal cancer needs to be analyzed in more research, and the extent of PORT CTV needs to be confirmed by more prospective studies.

## Conclusions

In conclusion, the most common type of postoperative recurrence of esophageal cancer was locoregional recurrence, especially with LNR. The LNR of the upper, middle, and lower thoracic segments were concentrated into three stations: No.104, No.106, and No.107. The rate of LNR outside the T-shaped PORT CTV we proposed was lower than 15%.

## Data Availability Statement 

The original contributions presented in the study are included in the article/supplementary material. Further inquiries can be directed to the corresponding author.

## Author Contributions

TC is responsible for research design, planning implementation, statistical analysis, and drafted the manuscript. HZ and TY collected important background information and carried out the data acquisition. YC and CL participated in study design and data acquisition. QZ and JZ carried out literature search and data aggregation. BL provided the theoretical proof and academic advice. WH is responsible for the topic selection, overall research guidance, and revision of the paper. All authors contributed to the article and approved the submitted version.

## Funding

We received funding for this study from the National Natural Science Foundation of China (81530060, 81773232, 81874224, 81671785, 81402538), the National Key Research and Develop Program of China (2016YFC0105106), the Key Research and Development Project of Shandong Province (2016GSF201123), the Foundation of Taishan Scholars (ts20120505, tsqn201909187, tsqn201909140), and Academic promotion program of Shandong First Medical University (2020RC002). This study was supported by National Natural Scientific Foundation (Grant No.81530060).

## Conflict of Interest

The authors declare that the research was conducted in the absence of any commercial or financial relationships that could be construed as a potential conflict of interest.

## Abbreviations

ESCC, esophageal squamous cell carcinoma; CTV, clinical target volume; PORT, postoperative radiotherapy; LNR, lymph node recurrence.
